# An online platform to increase access to gait rehabilitation for underserved Parkinson's disease communities

**DOI:** 10.1177/1877718X251410104

**Published:** 2025-12-30

**Authors:** Tamine TC Capato, Anouk Tosserams, Bastiaan R. Bloem, Jorik Nonnekes

**Affiliations:** 1 Department of Neurology, Centre of Expertise for Parkinson & Movement Disorders, Radboud University Medical Centre, Donders Institute for Brain, Cognition and Behaviour, Nijmegen, the Netherlands; 2 Department of Neurology, Movement Disorders Center, University of São Paulo, São Paulo, Brazil; 3 Department of Rehabilitation, Centre of Expertise for Parkinson & Movement Disorders, Radboud University Medical Centre, Donders Institute for Brain, Cognition and Behaviour, Nijmegen, The Netherlands; 4 Department of Rehabilitation, Sint Maartenskliniek, Nijmegen, The Netherlands

**Keywords:** Parkinson disease, gait, rehabilitation, vulnerable populations, rural health, Brazil, health services accessibility, internet-based intervention, telerehabilitation

## Abstract

Compensation strategies are a key element of gait rehabilitation in Parkinson's disease, ideally requiring involvement of specialized therapists. However, access to allied healthcare is not universally guaranteed. We evaluated an online platform to deliver compensation strategies for gait in 25 individuals with Parkinson's in rural Brazil. After three weeks of use, median patient-reported impact of gait impairment on daily activities [rated on visual analogue scale, 0–10] decreased from 7 to 4 (p < 0.001), without reported falls. This suggest that the platform is a safe and effective tool for supporting gait rehabilitation in underserved communities with limited access to healthcare services.

## Plain language summary title

Online support of PD gait rehabilitation

Plain language summary

Walking difficulties are common in people with Parkinson's Disease (PD). Use of compensation strategies can help to improve walking. However, not everyone has access to specialists who can guide the learning process. To address this, we delivered the compensation strategies via an online platform to 25 individuals with PD in rural Brazil. After three weeks of use, participants experienced less impact of walking problems in their daily lives. No falls were reported. This suggests that the platform is a safe and effective way to support gait rehabilitation in areas with limited access to healthcare services.

## Introduction

Gait impairment in Parkinson's disease (PD) is common and disabling: it significantly limits mobility, independence, and frequently results in falls.^1,2^ People with PD often use strategies to compensate for these walking difficulties. These so-called compensation strategies can be classified into seven main categories: 1) using external stimuli (external cues), 2) using self-generated stimuli (internal cues), 3) changing the balance requirements, 4) altering the mental state, 5) observation or visualizing walking (action observation or motor imagery), 6) adopting a new walking pattern, 7) alternatives to walking.^3^ Dedicated use of these compensation strategies has become an essential part of gait rehabilitation in PD, as conventional pharmacological treatment alone is often insufficient in ameliorating gait^4^ and balance deficits.^2,5,6^ The effect of these compensation strategies varies between individuals and is dependent on the context in which they are applied. Finding an optimal personal solution is therefore ideally supported by a specialized physiotherapist or occupational therapist, who can systematically search for appropriate strategies tailored to the individual needs of each patient.^7^

However, access to such specialized healthcare services is not universally available or guaranteed.^8,9^ Lack of equitable healthcare access poses a significant challenge in providing optimal care for people with PD in vulnerable populations.^10^ These populations may include persons living in developing countries, persons lacking health insurance or financial resources, or persons living in remote or rural areas with limited means of transportation. With the rising global burden of PD,^11–14^ innovative approaches to improve care provision in these underserved PD communities are warranted.

Digital technology solutions, such as online platforms and informative websites, have been utilized to improve management of PD.^15,16^ Here, we explore the safety and efficacy of using an online platform dedicated to compensation strategies for gait impairment to support rehabilitation in persons with PD living in underserved areas in Brazil, where access to allied healthcare services is scarce and varies significantly.^17,18^

## Methods

### Study population

This prospective single-arm cohort study was conducted in Brazil, in a rural area where access to allied healthcare services is limited, but where access to the internet is widespread.^19^ Participants with PD and self-reported disabling gait impairments who had never received physical or occupational therapy to improve their walking difficulties were included. Exclusion criteria comprised lack of internet access and cognitive impairment hampering the ability to provide informed consent or comply to the study protocol (Montreal Cognitive Assessment (MoCA) cut-off score of 18 points). Recruitment was achieved via the neurology outpatient clinics of the Movement Disorders Center of the University of São Paulo, Clinics Hospital (HC FMUSP), and its sisters hospitals, as well as through local PD support groups and social media advertisements (Ethics Committee approval – CONEP-7.171.656).

### Intervention

Participants were introduced to an online platform (www.walkingwithparkinson.com), which is dedicated to compensation strategies for gait impairment in PD. The existing Dutch and English versions were translated into Portuguese by a native speaker (www.andandocomparkinson.com.br). A research assistant facilitated the introduction, providing participants with the platform link, presenting an overview of the website's structure, and explaining how to access and navigate the video-based content. In addition, participants were also informed about the different categories of strategies and asked to test every strategy available on the platform. The Dutch and English versions of the online platform were developed in co-creation with people with PD. Its content is based on previous publications on compensation strategies.^3^ This platform contains background information about gait impairment in PD in plain language, and focuses on the framework of seven distinct categories of compensation strategies to overcome these gait impairments.^3^ The platform showcases 59 ready-to-use examples and illustrative patient videos of different compensation strategies, as well as a step-by-step tutorial on how to approach the search for suitable strategies systematically. Participants were instructed to carefully read the information provided on the platform about how it works, the step by step to find the strategies, and to test the different compensation strategies they came across for a period of three weeks in their own home environment, ideally accompanied by a partner, friend or family member. The compensation strategies were the same for all participants.

### Assessments

To assess the impact of using the online platform, pre- and post-intervention at-home assessments were performed by a trained research assistant. The same assistant performed both assessments to ensure consistency. All evaluations were carried out during the dopaminergic ON state and scheduled at the exact same time of day for both the pre- and post-intervention assessments. The MDS-UPDRS part III,^20^ plus the rapid turns test, were used for objective ascertainment of freezing of gait (FOG).^21^ Participants performed 360° turns from standstill in narrow quarters, at self-selected speed, with two leftward turns to and two rightward turns, in random order, as rapidly as possible. The occurrence and severity of FOG was also subjectively assessed using the New Freezing of Gait Questionnaire (NFOG-Q).^22^ The primary outcome measure was the self-reported impact of gait impairments on daily life activities expressed on a 0 (no impact) to 10 (maximum impact) visual analogue scale. Secondary outcomes included: the self-reported impact of freezing episodes on daily life activities expressed on a 0 (no impact) to 10 (maximum impact) visual analogue scale, number of compensation strategies known and used in daily life, self-esteem (measured by the Rosenberg Self Esteem Scale),^23^ fear of falling (Activities-Specific Balance Confidence Scale,^24^) quality of life (PDQ-39),^25^ and time spent frozen during a personalized gait trajectory in the patient's home (established through video annotation). The gait trajectory was assessed in the same location, both at baseline and post-intervention. Assessments were conducted in the patient's home environment. Patients self-reported contexts in which FOG previously occurred–for example, turning in the kitchen or bedroom, and walking through doorways–and these were included in the walking trajectory. Use of the person's own assistive devices (walkers or canes) was allowed. The post-intervention assessment of all primary and secondary outcomes was performed after three consecutive weeks of interacting with the online platform. Safety (i.e., occurrence of falls) and study adherence were monitored through weekly check-ins over the phone by a trained research assistant (see supplementary material for the questions asked).

### Statistical analysis

Descriptive statistical analyses were performed using IBM SPSS 29 (SPSS, Inc., Chicago, IL, USA). Independent samples T-tests and non-parametric independent Wilcoxon tests were performed to compare baseline vs. post-intervention outcome measures (α<0.05).

## Results

In total, 25 participants with PD were included (Table 1) from areas where access to allied healthcare services is limited, but where access to the internet is widespread.^19^ Table 2 illustrates the impact of the intervention on primary and secondary outcomes. The self-reported impact of gait impairments on daily life activities significantly decreased from baseline to post-intervention (from 7 to 4 on a 0–10 scale). Among the 18 participants with FOG, the self-reported impact of freezing episodes also significantly decreased following the intervention (from 6 to 3 on a 0–10 scale). FOG was rarely observed during the personalized gait trajectory; this did not differ between the pre- and post-intervention assessments. Other secondary outcomes involving balance confidence (ABC), quality of life (PDQ-39), and self-esteem (Rosenberg Self Esteem scale) demonstrated no significant improvement.

**Table 1.** Baseline characteristics.

	Total (n = 25)
Age (years)	60 [39–83]
Men (n (%))	13 (52%)
Time since PD diagnosis (years)	7 [2–27]
LEDD (mg)	900 [75–2198]
MDS-UPDRS part III, total score	40 [17–71]
Hoehn & Yahr stage	3 [2–4]
Presence of freezing of gait^a^ (n (%))	18 (72%)
NFOG-Q, total score^b^	14 [5–28]
Abnormal rapid turns test^a^ (n (%))	9 (50%)
MoCA, total score	26 [18–30]

Values represent median [range] unless otherwise specified.

^a^ Defined by a non-zero score on question 1 of the New Freezing of Gait Questionnaire (NFOG-Q).

^b^ Among participants with freezing of gait (n = 18).

PD = Parkinson's disease; LEDD = Levodopa Equivalent Daily Dose; MDS-UPDRS part III = Movement Disorders Society–Unified Parkinsons ´ Disease Rating Scale, motor score; NFOG-Q = New Freezing of Gait Questionnaire. MoCA = Montreal Cognitive Assessment.

**Table 2.** Outcomes at baseline and post-intervention.

	Baseline	Post-intervention	*p*
*Primary outcome*			
Impact of gait impairments on daily life activities, [0–10]	7 [2–10]	4 [0–10]	**<0**.**001**
*Secondary outcomes*			
Impact of freezing episodes on daily life activities,^a^ *[0–10]*	6 [0–10]	3 [0–9]	**0**.**022**
Percentage of time spent frozen during a personalized gait trajectory,^a^ (%)	0 [0–5]	0 [0–4]	0.127
Categories of compensation strategies known, *[0–7]* (n)	0 [0–0]	3 [1–7]	**<0**.**001**
Individual strategies known (n)	0 [0–0]	13 [1–55]	**<0**.**001**
Categories of compensation strategies used in daily life, *[0–7]* (n)	0 [0–0]	2 [0–6]	**<0**.**001**
Individual strategies used in daily life (n)	0 [0–0]	2 [0–7]	**<0**.**001**
Rosenberg Self Esteem Scale, total score *[0–30]*	21 [14–29]	21 [13–32]	0.546
Activities-specific Balance Confidence Scale, total score *[0–100]*	67 [28–84]	72 [22–96]	0.091
Parkinson's Disease Questionnaire-39, total score *[0–100]*	37 [10–74]	38 [10–90]	0.973

Values represent median [range].

*P* values <0.05 are indicated in bold.

^a^ Among participants with freezing of gait (n = 18).

The study was well-received and appreciated by 92% of the participants, of which 40% asked someone for help using the online platform (elderly with severe motor and/or mild cognitive impairments). Only 16% of participants reported some Wi-Fi problems, which did not influence the intervention compliance. Most participants were able to learn and implement new strategies by interacting with the platform, as reflected by an overall increase in the number of strategies known and used in daily life; 68% of participants had implement one or more strategies in their daily routines.

No falls or other adverse events were reported by participants during the three-week study period.

## Discussion

This study evaluated the usefulness of an online platform with a menu of compensation strategies for gait impairment in PD, with the aim of supporting gait rehabilitation. Most participants successfully learned and applied compensation strategies to improve gait in their home setting. Our findings suggest that online education on compensation strategies can improve the perceived impact of gait impairment on daily life activities. At baseline, none of the participants knew any of the compensation strategies, stressing the need for education on this topic. Previous studies from our research group also identified this lack of knowledge about compensation strategies.^7^

Despite the promising findings of our study, several limitations should be considered. First, the primary outcome relied on self-reported impact scores, which may be influenced by personal bias, motivation or expectations. We did assess the percentage of time spent frozen during a personalized gait trajectory using video annotation as a secondary outcome of efficacy, however because FOG rarely occurred during the baseline gait trajectory, we were unable to infer the efficacy of the application of the strategies at post-intervention. More detailed objective measures (e.g., using wearable sensors)^16,26^ could have provided a more precise evaluation of potential gait changes at home as a result of interacting with the online platform. However, given the remote setting of the study, and our aim to explore the usefulness of an online educational platform in underserved populations, the use of participant-reported outcome measures was deemed to be the most feasible research strategy. Second, we did not assess whether the subjective improvements in gait were sustained over time, or whether people with PD were able to retain their learned strategies after the study period. This would be particularly relevant in the face of cognitive impairment, and other non-motor symptoms, which are often present in persons with PD and significant gait impairment.^1,2,27^ Of note, presence of cognitive impairment may also hamper one's ability to successfully navigate a digital educational platform in the first place. Finally, we acknowledge that innovative interventions such as dedicated education through an online platform require some level of digital literacy. In our study, participants with low digital literacy used the platform with the support of their family members, furthermore they were able to ask questions regarding the platform during the weekly check-ins with the researcher over the phone. Therefore their level of digital literacy was deemed unlikely to affect the success of the online platform. These results cannot automatically be extrapolated to underserved populations at large, particularly persons without such a support system present. However, access to online services has become abundant worldwide, also in developing countries, and the uptake of technology will only increase further in coming years, so we expect to be able to serve a sizeable proportion of the global PD population with this new online service.^9,15^

A potential concern is that certain compensation strategies may aggravate gait impairments^7^ in some individuals. However, no falls or other adverse events were reported during the three-week period during which participants interacted with the online platform. This suggests that unsupervised use of an online platform to explore gait compensation strategies in the home setting may be a safe option to support gait rehabilitation in individuals with PD who have limited access to (specialized) allied healthcare services. Earlier studies on unsupervised at-home gait interventions^28–30^ and online interventions^31,32^ in persons with PD yielded similar results in terms of safety. While optimal strategies for remote education and intervention delivery have yet to be established globally,^33,34^ our platform contributes to the democratization of such interventions by reaching underserved communities. Moreover, it offers health professionals an additional tool to address the global challenge of delivering accessible and equitable rehabilitation care in PD.^34–36^ Our platform can easily be translated and culturally adapted to other languages.^37^

Overall, it appears that online educational platforms offer the opportunity to support gait rehabilitation when access to allied healthcare services is limited, provided that persons have access to internet and are sufficiently digitally literate. A continued global effort is warranted to provide innovative solutions to ameliorate access to healthcare services in vulnerable PD communities, and to facilitate optimal care for every person living with PD worldwide.

## References

[1] Nonnekes J , et al. Neurological disorders of gait, balance and posture: a sign-based approach. Nat Rev Neurol 2018; 14: 183–189. 29377011 10.1038/nrneurol.2017.178

[2] Mirelman A , et al. Gait impairments in Parkinson's disease. Lancet Neurol 2019; 18: 697–708. 30975519 10.1016/S1474-4422(19)30044-4

[3] Nonnekes J , et al. Compensation strategies for gait impairments in Parkinson disease: a review. JAMA Neurol 2019; 76: 718–725. 30907948 10.1001/jamaneurol.2019.0033

[4] Nonnekes J , et al. Freezing of gait: a practical approach to management. Lancet Neurol 2015; 14: 768–778. 26018593 10.1016/S1474-4422(15)00041-1

[5] Camicioli R , et al. Prevention of falls in Parkinson's disease: guidelines and Gaps. Mov Disord Clin Pract 2023; 10: 1459–1469. 37868930 10.1002/mdc3.13860 PMC10585979

[6] Lamont RM , et al. Falls in people with Parkinson's disease: a prospective comparison of community and home-based falls. Gait Posture 2017; 55: 62–67. 28419875 10.1016/j.gaitpost.2017.04.005

[7] Tosserams A , et al. Perception and use of compensation strategies for gait impairment by persons with Parkinson disease. Neurology 2021; 97: e1404–e1412. 10.1212/WNL.0000000000012633 PMC8520387 34497067

[8] Stocchi F Bloem BR . Move for change part II: a European survey evaluating the impact of the EPDA charter for people with Parkinson's disease. Eur J Neurol 2013; 20: 461–472. 23034057 10.1111/j.1468-1331.2012.03876.x PMC3593160

[9] Schiess N , et al. Six action steps to address global disparities in Parkinson disease: a world health organization priority. JAMA Neurol 2022; 79: 929–936. 35816299 10.1001/jamaneurol.2022.1783

[10] Bhidayasiri R Kalia LV Bloem BR . Tackling Parkinson's disease as a global challenge. J Parkinsons Dis 2023; 13: 1277–1280. 38143374 10.3233/JPD-239005 PMC10741319

[11] Dorsey ER , et al. The emerging evidence of the Parkinson pandemic. J Parkinsons Dis 2018; 8: S3–S8. 10.3233/JPD-181474 PMC6311367 30584159

[12] Dorsey ER , et al. Projected number of people with Parkinson disease in the most populous nations, 2005 through 2030. Neurology 2007; 68: 384–386. 17082464 10.1212/01.wnl.0000247740.47667.03

[13] Rocca WA . The future burden of Parkinson's disease. Mov Disord 2018; 33: 8–9. 28782890 10.1002/mds.27114 PMC5771661

[14] Rossi A , et al. Projection of the prevalence of Parkinson's disease in the coming decades: revisited. Mov Disord 2018; 33: 156–159. 28590580 10.1002/mds.27063 PMC5720940

[15] Bloem BR , et al. Integrated and patient-centred management of Parkinson's disease: a network model for reshaping chronic neurological care. Lancet Neurol 2020; 19: 623–634. 32464101 10.1016/S1474-4422(20)30064-8 PMC9671491

[16] Espay AJ , et al. A roadmap for implementation of patient-centered digital outcome measures in Parkinson's disease obtained using mobile health technologies. Mov Disord 2019; 34: 657–663. 30901495 10.1002/mds.27671 PMC6520192

[17] Schlickmann TH , et al. Prevalence, distribution and future projections of Parkinson disease in Brazil: insights from the ELSI-Brazil cohort study. Lancet Reg Health Am 2025; 44: 101046. 40230590 10.1016/j.lana.2025.101046 PMC11995787

[18] Oliveira ECT , et al. Difficulties in accessing health services among the elderly in the city of São Paulo-Brazil. PLoS One 2022; 17: e0268519. 10.1371/journal.pone.0268519 PMC9119537 35588124

[19] IBGE. Acesso à Internet e à televisão e posse de telefone móvel celular para uso pessoal 2021, in Diretoria de Pesquisas, Coordenação de Pesquisas por Amostra de Domicílios, Pesquisa Nacional por Amostra de Domicílios Contínua. 1. Rio de Janeiro, Brazil: Instituto Brasileiro de Geografia e Estatística (IBGE): online, 2022. https://biblioteca.ibge.gov.br/index.php/biblioteca-catalogo?view=detalhes&id=2101963

[20] Goetz CG , et al. Movement disorder society-sponsored revision of the unified Parkinson's disease rating scale (MDS-UPDRS): scale presentation and clinimetric testing results. Mov Disord 2008; 23: 2129–2170. 19025984 10.1002/mds.22340

[21] Snijders AH , et al. Freezer or non-freezer: clinical assessment of freezing of gait. Parkinsonism Relat Disord 2012; 18: 149–154. 21968033 10.1016/j.parkreldis.2011.09.006

[22] Nieuwboer A , et al. Reliability of the new freezing of gait questionnaire: agreement between patients with Parkinson's disease and their carers. Gait Posture 2009; 30: 459–463. 19660949 10.1016/j.gaitpost.2009.07.108

[23] Rosenberg M . Society and the adolescent self-image. Princeton, NJ: Princeton University Press., 1965.

[24] Powell LE Myers AM . The activities-specific balance confidence (ABC) scale. The Journals of Gerontology: Series A 1995; 50A: M28–M34. 10.1093/gerona/50a.1.m28 7814786

[25] Peto V , et al. The development and validation of a short measure of functioning and well being for individuals with Parkinson's disease. Qual Life Res 1995; 4: 241–248. 7613534 10.1007/BF02260863

[26] Capato TTC , et al. Assisted technology in Parkinson's disease gait: what's up? Arq Neuropsiquiatr 2024; 82: 1–10. 10.1055/s-0043-1777782 PMC10890908 38395424

[27] Still A , et al. Relationships between physical activities performed under free-living conditions and non-motor symptoms in people with Parkinson's: a systematic review and meta-analysis. Clin Rehabil 2024; 38: 1534–1551. 39175369 10.1177/02692155241272967 PMC11528973

[28] Putzolu M , et al. Home-based exercise training by using a smartphone app in patients with Parkinson’s disease: a feasibility study. Front Neurol 2023; 28: 14. 10.3389/fneur.2023.1205386 PMC10338039 37448748

[29] Nieuwboer A , et al. Cueing training in the home improves gait-related mobility in Parkinson's disease: the RESCUE trial. J Neurol Neurosurg Psychiatry 2007; 78: 134–140. 17229744 10.1136/jnnp.200X.097923 PMC2077658

[30] Morris ME , et al. A home program of strength training, movement strategy training and education did not prevent falls in people with Parkinson's disease: a randomised trial. J Physiother 2017; 63: 94–100. 28342682 10.1016/j.jphys.2017.02.015

[31] Morris ME , et al. Online dance therapy for people with Parkinson's disease: feasibility and impact on consumer engagement. Neurorehabil Neural Repair 2021; 35: 1076–1087. 34587834 10.1177/15459683211046254

[32] Domingos J , et al. An online dual-task cognitive and motor exercise program for individuals with Parkinson disease (PD3 move program): acceptability study. JMIR Aging 2022; 5: e40325. 10.2196/40325 PMC9816951 36548037

[33] Shin JH Kim HJ Jeon B . Important advances in movement disorders research in 2023. Lancet Neurol 2024; 23: 20–22. 38101889 10.1016/S1474-4422(23)00461-1

[34] Cilia R , et al. Delivery of Allied Health Therapies to People with Parkinson's Disease in Africa. J Parkinsons Dis 2024; 14: S227–S239. 10.3233/JPD-230262 PMC11380278 38143371

[35] Cilia R , et al. Natural history of motor symptoms in Parkinson's disease and the long-duration response to levodopa. Brain 2020; 143: 2490–2501. 32844196 10.1093/brain/awaa181 PMC7566883

[36] Visser LM , et al. Do online communities change power processes in healthcare? Using case studies to examine the use of online health communities by patients with Parkinson's disease. BMJ Open 2016; 6: e012110. 10.1136/bmjopen-2016-012110 PMC5128832 27821596

[37] Domingos JMM , et al. The European physiotherapy guideline for Parkinson's disease: translation for non-English speaking countries. J Neurol 2021; 268: 214–218. 32761506 10.1007/s00415-020-10132-x

